# Genomic analysis of post-mating changes in the honey bee queen (*Apis mellifera*)

**DOI:** 10.1186/1471-2164-9-232

**Published:** 2008-05-19

**Authors:** Sarah D Kocher, Freddie-Jeanne Richard, David R Tarpy, Christina M Grozinger

**Affiliations:** 1 Department of Genetics, North Carolina State University, Raleigh, NC, USA; 2 Department of Entomology, North Carolina State University, Raleigh, NC, USA; 3 W.M. Keck Center for Behavioral Biology, North Carolina State University, Raleigh, NC, USA

## Abstract

**Background:**

The molecular mechanisms underlying the post-mating behavioral and physiological transitions undergone by females have not been explored in great detail. Honey bees represent an excellent model system in which to address these questions because they exhibit a range of "mating states," with two extremes (virgins and egg-laying, mated queens) that differ dramatically in their behavior, pheromone profiles, and physiology. We used an incompletely-mated mating-state to understand the molecular processes that underlie the transition from a virgin to a mated, egg-laying queen. We used same-aged virgins, queens that mated once but did not initiate egg-laying, and queens that mated once and initiated egg-laying.

**Results:**

Differences in the behavior and physiology among groups correlated with the underlying variance observed in the top 50 predictive genes in the brains and the ovaries. These changes were correlated with either a behaviorally-associated pattern or a physiologically-associated pattern. Overall, these results suggest that the brains and the ovaries of queens are uncoupled or follow different timescales; the initiation of mating triggers immediate changes in the ovaries, while changes in the brain may require additional stimuli or take a longer time to complete. Comparison of our results to previous studies of post-mating changes in *Drosophila melanogaster *identified common biological processes affected by mating, including stress response and alternative-splicing pathways. Comparison with microarray data sets related to worker behavior revealed no obvious correlation between genes regulated by mating and genes regulated by behavior/physiology in workers.

**Conclusion:**

Studying the underlying molecular mechanisms of post-mating changes in honey bee queens will not only give us insight into how molecular mechanisms regulate physiological and behavioral changes, but they may also lead to important insights into the evolution of social behavior. Post-mating changes in gene regulation in the brains and ovaries of honey bee queens appear to be triggered by different stimuli and may occur on different timescales, potentially allowing changes in the brains and the ovaries to be uncoupled.

## Background

Mating causes extensive short- and long-term modifications of physiology and behavior in females. In insects, mated females often become refractory to additional mating, their ovaries become activated, they form mature eggs, and they initiate egg-laying and/or foraging behavior [1-3]. However, there have been few studies that examine the molecular mechanisms underlying these post-mating changes, and all of these have been carried out using *Drosophila melanogaster *[1,3,4].

The queen honey bee (*Apis mellifera*) provides an excellent model to study the genes that regulate these behavioral and physiological transitions. The mating process in honey bees has been very well-characterized, and the behavioral and physiological differences between virgin and mated queens are extensive and have been well studied [5,6]. Furthermore, because the mating process can also be quite prolonged, it is possible to analyze intermediate states – in addition to virgin and mated, laying queens, we can monitor behavior, physiology, and gene-expression patterns in mated queens that have not yet initiated egg-laying. With the release of the honey bee genome [6], we now have the tools available to elucidate the genetic changes associated with the observed changes in behavior and physiology.

Mating in honey bee queens can be a protracted process (reviewed in [6]). A queen bee reaches sexual maturity when she is 5–10 days old, at which point she initiates mating flights. She will take one to three mating flights on subsequent days, and mate with an average of 12 drones throughout the course of these flights [8]. The majority of the semen collected by the queen is excreted within 24 hours after insemination, and a proportion of each male's sperm is stored in her spermatheca. A fully inseminated queen carries approximately 5–7 million sperm [9]. These averages vary tremendously among individuals, populations, and other bee species within the genus *Apis *[10]. Once the queen completes the mating process, she will initiate egg-laying behavior within a few days, and then will never mate again during her 1–5 year lifespan.

After mating, a queen undergoes considerable physiological and behavioral changes. Her ovaries (previously in a state of arrested development) complete the final stages of maturation as her ovarioles increase in size and the eggs become vitellogenic and reach, maturity [11]. The queen's pheromone profile also changes dramatically [12-14], causing workers to surround the queen in a retinue response, antennating and licking her. Major changes occur in the brain as well. Fahrbach et al. [15] found that following mating, the Kenyon cells of the mushroom bodies decrease by 30% while the neuropil of the mushroom bodies increases by 25–50%. Levels of dopamine and N-acetyldopamine also decrease following mating [16]. Mating also causes profound behavioral changes. Virgins are phototactic and take mating flights, while mature, mated queens remain in their colonies and lay eggs [6].

In order to disentangle the molecular pathways regulating these various components of the post-mating response, we correlated changes in gene-expression patterns in the brains and ovaries to behavioral and physiological changes in three distinct types of queens: virgins (n = 6), mated queens that have not yet initiated egg-laying (subsequently denoted as "mated queens", n = 5), and mated queens that have begun laying eggs (subsequently denoted as "laying queens", n = 4). Virgin queens and laying queens are the two extreme cases encompassing the complete post-mating response, while the second group (mated queens) represents an intermediate phenotype. These queens are considered to be "incompletely-mated", because they have not yet completed the full suite of behavioral and physiological transitions associated with mating in honey bees. It is also important to note that queens in this group were heterogeneous in terms of flight behavior; some attempted to take additional mating flights, while others were not observed to do so. Four queens from each group were used for the microarray study. Our results demonstrate that behavioral and physiological post-mating changes can be uncoupled in the mated group, and that these changes can be associated with specific gene expression patterns. Furthermore, we compared our results to previous microarray studies in *Drosophila *[3] to determine if genomic changes due to mating were similar in these different insect species, but found that few genes were similarly regulated between these studies. Finally, we found no obvious correlation between genes regulated by mating and genes regulated by behavior or physiology in workers.

## Results

### Behavioral Data

**Figure 1:**
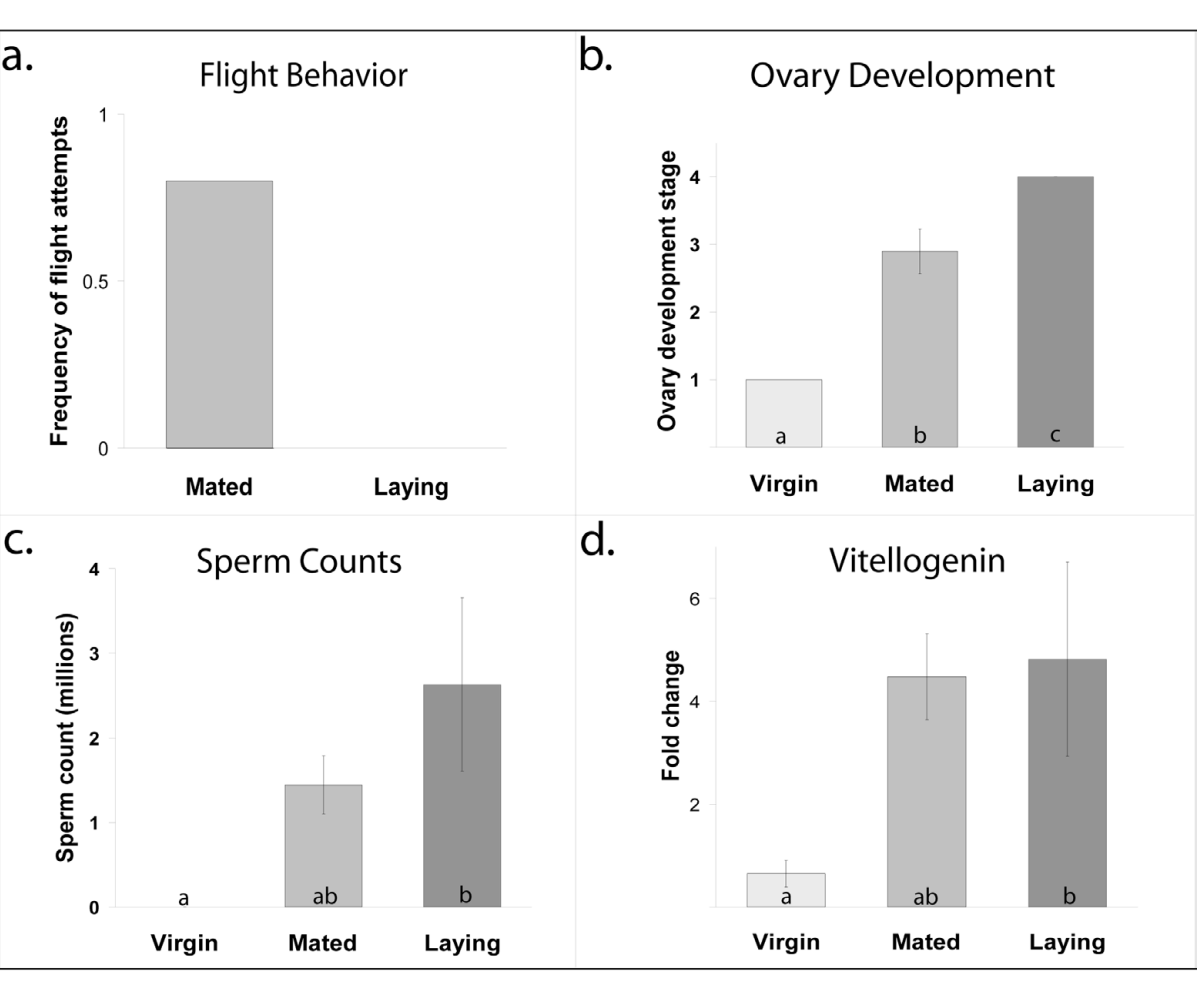
**The effects of mating on queen behavior and physiology**. (a) Non-virgin queens were allowed only a single mating flight. All subsequent flight attempts were monitored. None of the queens initiating egg-laying attempted to fly following mating. (b) Ovary development scores ranging from 1 (no development) to 4 (fully developed with mature eggs present) were assigned to each queen. Laying queens all had fully-developed ovaries with mature eggs. Mated queens initiated ovary development while virgins did not (Kruskall-Wallis rank sums, p = 0.0016). Of the mated queens, 1/5 had an ovary development score of 2, 1/5 had a score of 2.5, 2/5 received a score of 3, and 1/5 had a score of 4. (c) Sperm counts demonstrated that mated queens were intermediate for sperm storage in their spermathecae (Kruskall-Wallis rank sums, p = 0.004, nonparametric Tukey-Kramer HSD, p < 0.05). (d) *Vitellogenin *levels in the fat bodies were significantly different between virgins and laying queens (Kruskall-Wallis rank sums, p = 0.018, nonparametric Tukey-Kramer HSD, p < 0.05). Abdominal *vitellogenin *levels were positively correlated with ovary development (R^2 ^= 0.46, p = 0.006).

Flight attempts of queens that had been allowed to take only a single mating flight were monitored. When queens were 5 days old, colony entrances were opened so they could take mating flights. Of the 10 queens allowed to fly, 9 mated within 2 days. Four of these initiated egg-laying within five days, while the remainder did not. None of the queens that initiated egg-laying were observed to attempt additional mating flights. Of the five remaining queens (the mated queens), three attempted to take additional mating flights (Figure 1a). Two of these were represented in the mated group in the arrays. The remaining two array samples from this group were queens that did not attempt to take additional mating flights.

### Ovary Development

The ovaries were dissected from the queens and assigned a development score ranging from 1 (no development) to 4 (fully developed ovaries with mature eggs). Ovary development differed among all three groups (Kruskall-Wallis rank sums, p < 0.0016, nonparametric Tukey-Kramer HSD, p < 0.05). Virgin queens (n = 6) had not yet initiated ovary development (average score = 1). Mated queens (n = 5) had initiated ovary development (average score = 2.9), however none of these queens had fully developed ovaries. Laying queens (n = 4) all had mature eggs present in their ovaries (average score = 4) (Figure 1b).

### Sperm Counts

The number of sperm stored in the spermathecae of the queens was counted after collection. All of the virgin queens were examined as well to verify that they had not mated. Sperm numbers varied significantly among groups (Kruskall-Wallis rank sums, p = 0.004). Means were compared using a nonparametric Tukey-Kramer HSD test. Laying queens and virgins had significantly different numbers of sperm (p < 0.05), while sperm numbers in mated queens were at intermediate levels, and not significantly different from virgins or laying queens (Figure 1c).

### Vitellogenin levels

Vitellogenin is an egg-yolk protein that is synthesized in the fat bodies and transported into the eggs during maturation. Quantitative, real-time PCR revealed that levels of *vitellogenin *varied significantly among groups (n = 4, all groups, Kruskall-Wallis rank sums, p = 0.018). Means were compared using a nonparametric Tukey-Kramer HSD. There was a significant difference between virgins and laying queens in *vitellogenin *RNA levels present in the abdominal fat bodies (p < 0.05), but there was no significant difference in *vitellogenin *RNA levels between mated and laying queens or mated and virgin queens (Figure 1d). Levels of *vitellogenin *were positively correlated with ovary development (R^2 ^= 0.46, p = 0.0058).

### Pheromone Profiles

**Figure 2:**
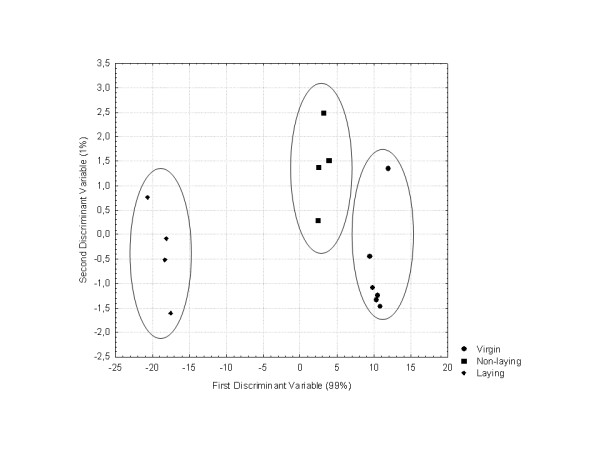
**Queen Pheromone Profiles**. Discriminant analysis of queen mandibular gland chemical profiles of virgins, mated, and laying queens. Chemical composition of the mandibular gland extracts of virgin, mated and laying queens were analysed using gas chromatography. Discriminant analysis of mandibular extract of queens was based on the relative proportion of the chemical compounds (F(18,6) = 28.44, p = 0.0002).

Gas chromatography of the queen mandibular glands of the three groups of queens (virgin, mated and laying queens) revealed a significant difference in the overall profile (F(18,6) = 28.44, p = 0.0002, Figure 2). Two discriminant variables explained 100% of the variation. While the three types of queens clustered into distinct groups, the greatest difference occurred between the virgin/mated queen clusters and the laying queens, suggesting that the pheromone profiles of the mated queens were in the early stages of transition (Figure 2). Relative quantities of the mandibular gland compounds are included in Additional file 1.

### Brain Gene Expression Profiles

**Figure 3:**
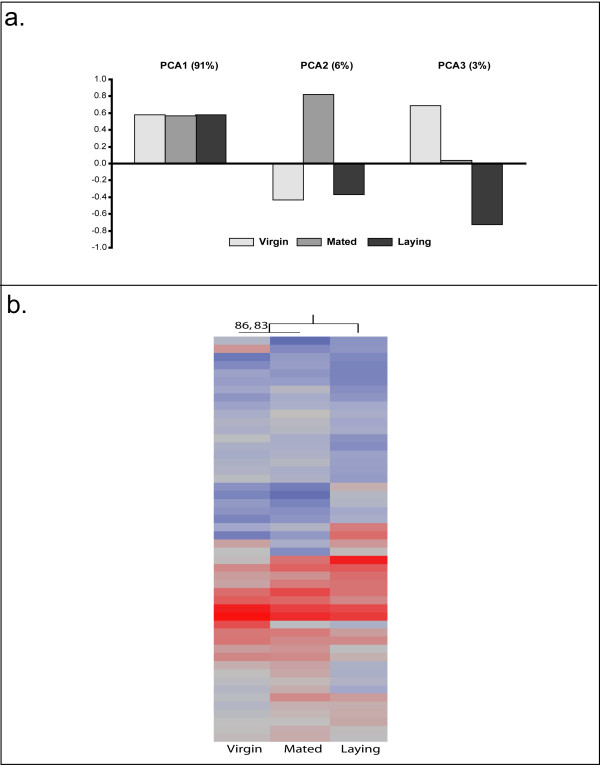
**Analysis of Brain Gene Expression**. a. Principal component analysis of the significantly-regulated genes revealed three major patterns of changes occurring within the brain. The first principal component accounts for 91% of the variation and shows little difference between the three groups, while the second principal component accounts for 6% of the variation, with the virgin and laying groups resembling each other and the mated group as the extreme. The final principal component accounts for 2% of the overall variation, and demonstrates that virgin and laying queens are at two extremes with mated queens intermediate. b. Hierarchical clustering analysis was employed to determine if there was a significant clustering structure among the 50 most-predictive transcripts in the brain. Virgin and mated queens grouped together with laying queens as the outgroup. This grouping is supported by an "approximately-unbiased" p-value of 86 and a bootstrap value of 83, and shares the same pattern observed in behavior and mandibular gland profiles.

Statistical analysis revealed that out of 10,468 transcripts included in the analysis, 971 were differentially expressed in the brain among our queen groups (n = 4, all groups, FDR < 0.05). Hierarchical clustering analysis using all of the significant genes grouped virgin queens with laying queens [see Additional file 6]. Principal component analysis was performed to determine if there were any specific underlying expression patterns in the significantly regulated genes across all three groups (Figure 3a). The first principal component accounts for 91% of the variation and shows little difference between the three groups, while the second principal component accounts for 6% of the variation, with the virgin and laying groups resembling each other and the mated group as the extreme. The final principal component accounts for 2% of the overall variation, and demonstrates that virgin and laying queens are at two extremes with mated queens intermediate.

The top 50 predictive genes were identified using the class prediction function in GeneSpring. Leave-one-out cross-validation using these genes correctly identified 11/12 samples – one virgin was unpredicted. Clustering analysis grouped virgin and mated queens together, with an "approximate-unbiased" p-value score of 86, and a bootstrap support of 83 (Figure 3b).

Gene ontology analysis (Table 1) using DAVID [17] reveals that among the significantly regulated genes, there is a significant overrepresentation of genes involved in protein folding (p < 0.004), protein catabolism (p < 0.016), and stress response (p < 0.034). Several of these genes were part of three pathways: a purine metabolic pathway, proteasome structure, and pyrimidine metabolism. The 50 predictive genes were input into DAVID to identify overrepresented biological functions, but no groups survived the Benjamini correction. A table of these genes is provided in the supplementary data [see Additional file 2].

**Table 1:** Overrepresented GO Biological Processes in the brain

**GO Biological Process**	**Count**	**%**	**p-value**
protein folding	19	2.64%	0.004
ubiquitin-dependent protein catabolism	12	1.67%	0.016
modification-dependent protein catabolism	12	1.67%	0.022
M phase	28	3.89%	0.025
cellular metabolism	315	43.81%	0.025
proteolysis during cellular protein catabolism	12	1.67%	0.032
cellular protein catabolism	12	1.67%	0.032
response to stress	29	4.03%	0.034
catabolism	33	4.59%	0.034
nuclear migration	5	0.70%	0.036

Top 10 significant GO biological processes that were overrepresented in expression profiling of the brains. One major pathway, involved in purine metabolism, was significantly overrepresented by our data set, as well as genes associated with metabolism and stress response.

### Ovary Gene Expression Profiles

**Figure 4:**
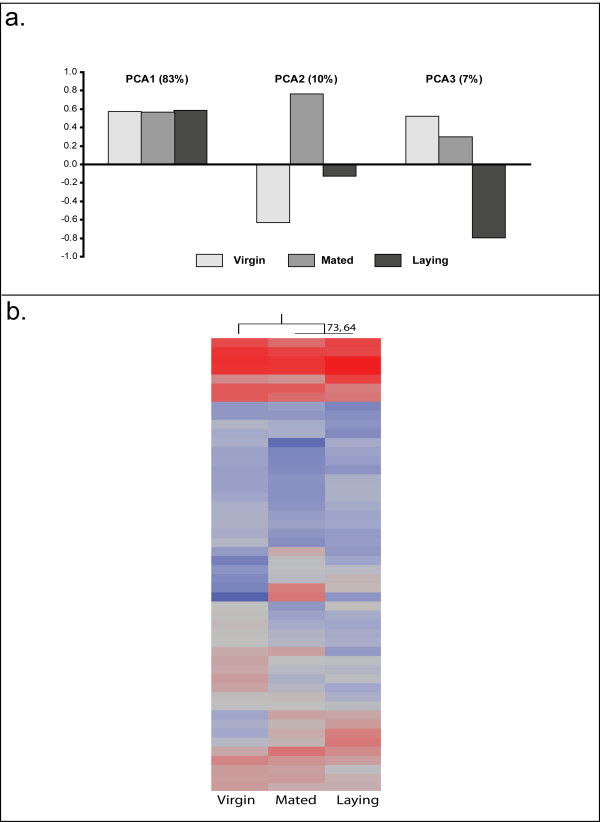
**Analysis of Ovary Gene Expression**. a. Principal components analysis of transcript profiling in the ovaries demonstrated similar patterns as the brain. The first principal component accounts for 83% of the variation, and shows little differences among groups. The second principal component accounts for 10% of the overall variation and demonstrates that mated queens represent an extreme phenotype. The final principal component explains 7% of the variation, and identifies laying queens as the outgroup. b. Hierarchical clustering of the 50 most-predictive transcripts in the ovaries reveals that mated and laying queens have transcriptional profiles more similar to each other than to virgins. This grouping is supported by an "approximately-unbiased" p-value of 73 and a boostrap value of 64, and reflects the same patterns observed in ovary development and *vitellogenin *levels.

Statistical analysis revealed that out of the 7,377 transcripts included in the analysis, 366 were differentially expressed in the ovaries among all groups (n = 4, all groups, FDR < 0.05). Hierarchical clustering analysis using all regulated genes grouped virgin and laying queens together for overall gene expression patterns [see Additional file 7]. Principal components analysis of all regulated transcripts in the ovaries demonstrated similar patterns as the brain (Figure 4a). The first principal component accounts for 83% of the variation, and shows little differences among groups. The second principal component accounts for 10% of the overall variation and demonstrates that mated queens represent an extreme phenotype. The final principal component explains 7% of the variation, and identifies laying queens as the outgroup.

The top 50 predictive genes were identified using the class prediction function in GeneSpring. Leave-one-out cross-validation using these genes correctly identified 8/12 samples. All virgins were correct. Two laying queens were unpredicted, and two mated queens were unpredicted. Hierarchical clustering of the 50 predictive genes grouped mated and laying queens together, with an "approximately-unbiased" p-value score of 73 and a bootstrap value of 64 (Figure 4b).

Gene-ontogeny analysis (Table 2) of the 366 significantly regulated genes revealed that they were largely involved in cell division (p < 0.002), gametogenesis (p < 0.002), reproduction (p < 0.003), and oogenesis (p < 0.028). The 50 predictive genes were input into DAVID to identify overrepresented biological functions, but no groups survived the Benjamini correction. A table of these genes is provided in the supplementary data [see Additional file 3].

**Table 2:** Overrepresented GO Biological Processes in the ovaries

**GO Biological Process**	**Count**	**%**	**p-value**
cell division	11	4.31%	0.002
gametogenesis	22	8.63%	0.002
sexual reproduction	22	8.63%	0.003
development	51	20.00%	0.003
mitosis	14	5.49%	0.003
M phase of mitotic cell cycle	14	5.49%	0.003
protein complex assembly	10	3.92%	0.005
cell differentiation	23	9.02%	0.005
reproduction	22	8.63%	0.006
M phase	15	5.88%	0.007

Top 10 significant GO biological processes overrepresented in ovarian transcriptional profiling. Due to the large number of overrepresented terms, duplicate groups and groups with less than 15 genes were removed from this table. Several groups involved in reproduction, oogenesis, and development appear in this list.

There were 26 transcripts that were regulated in both the brains and the ovaries. A list of these genes is included in Additional file 4.

### Comparative Analyses

#### Changes in transcript abundance in honey bees and *Drosophila*

We compared our results from the brain transcriptional profiling to the results of a study published by McGraw et.al. [3]. In this study, whole-body expression differences in virgin and mated female *Drosophila *were compared 1–3 hours post-mating. Of the genes expressed in our brain arrays, 336 were also found on the *Drosophila *arrays performed by McGraw et al. [3]. Only 21 genes from this list of 336 were significantly regulated by mating in both bees and *Drosophila *[see Additional file 5]. In the 21 transcripts differentially expressed in both data sets, several GO biological processes were overrepresented, such as stress response, alternative splicing, protein binding, and tissue development (Table 3).

**Table 3:** Biased expression of gene categories significantly regulated by mating in both *Drosophila *and *Apis mellifera*

**GO Biological Process**	**Count**	**%**	**p-value**
response to stress	5	19.23%	0.002
alternative splicing	5	19.23%	0.005
protein binding	16	61.54%	0.007
response to hypoxia	2	7.69%	0.009
calcium ion binding	4	15.38%	0.010
mitotic spindle checkpoint	2	7.69%	0.018
spindle checkpoint	2	7.69%	0.018
anterior/posterior axis specification	3	11.54%	0.018
anterior/posterior pattern formation	3	11.54%	0.022
tissue development	4	15.38%	0.024
mitotic checkpoint	2	7.69%	0.025
calcium binding	2	7.69%	0.028
axis specification	3	11.54%	0.030
cell cycle checkpoint	2	7.69%	0.031
acetylation	2	7.69%	0.032
mesoderm development	3	11.54%	0.040
ventral cord development	2	7.69%	0.044
physiological process	17	65.38%	0.047
splice variant	5	19.23%	0.047
cytoplasm	6	23.08%	0.048

We compared our results to the results of a study published by McGraw et.al. (2004) that identified changes in gene expression in *Drosophila *females 1–3 hours after mating. Of the genes on our honey bee arrays, 336 were also found on the arrays used by McGraw et.al. (2004) [3]. Only 21 genes from this list were significantly regulated by mating in both bees and *Drosophila*. Several GO biological processes were overrepresented among the 21 transcripts differentially expressed in both data sets, such as: stress response, alternative splicing, protein binding, and tissue development.

#### Correlations with worker honey bee development, QMP-response, and reproduction

Previous microarray studies identified 221 genes that were expressed at significantly different levels in the brains of reproductive and sterile worker bees [18]. Of these 221 genes, 64 genes were expressed at high enough levels in our brain samples to be included in our analysis. Of these, only two were significantly differentially expressed [see Additional file 5].

A study published by Whitfield et.al [19] identified sets of genes regulated by methoprene (an analog of juvenile hormone) in worker bees. In queens, juvenile hormone levels are lower in mated than newly emerged virgin queens [20], but juvenile hormone levels do not change after mating [20] and do not appear to be directly involved in ovary development (reviewed in [21]). To examine whether levels of juvenile hormone may be involved in producing the changes in gene expression associated with mating, we compared our list of significantly regulated genes in the brain to these methoprene-regulated genes [19]. Of the 306 genes that were both upregulated by methoprene and expressed in the brains of our queens, only 26 (9.47%) were significantly regulated by mating [see Additional file 5]. Of the 207 genes that were both downregulated by methoprene and present in our study, only 19 (9.17%) were significantly regulated by mating [see Additional file 5]. Thus, there does not appear to be any particular biased involvement of methoprene-regulated genes in the post-mating changes.

Finally, we examined whether genes regulated by queen mandibular pheromone (QMP) in the brains of workers were regulated by the mating process in the brains of queens. QMP regulates many aspects of worker behavior and physiology [22], including inhibiting ovary development [23], and thus QMP-regulated genes may be associated with reproduction in queens. We compared our data to a study published by Grozinger et.al. [24] and found no obvious association of mating with QMP-regulated genes in workers. Of the 491 genes which were both upregulated by QMP and present in our analysis, only 43 (8.75%) were significantly regulated by mating, while only 32 (6.39%) of the 501 genes that were both downregulated by QMP and present in our analysis, were significantly regulated [see Additional file 5].

#### Correlation with genes associated with worker division of labor

One of the major theories in the evolution of eusocial behavior is the concept that genes involved in reproduction may have been co-opted for use in division of labor [25-27]. In honey bees, worker division of labor involves a behavioral maturation process, in which young bees care for brood (nurse bees) while older bees become foragers [6]. To determine if genes associated with worker division of labor are also preferentially associated with reproductive state in queens, we examined the brain expression patterns of sets of genes previously identified by Whitfield et.al. [28] as expressed at significantly higher levels in the brains of nurses vs. foragers (nursing-associated) or expressed at significantly higher levels in the brains of foragers vs. nurses (foraging-associated). 599 nursing-associated genes were expressed in the brains of our queens, but only 53 (8.84%) were significantly regulated by mating [see Additional file 5]. Similarly, 401 foraging-associated genes were expressed in our data set, but only 30 (7.48%) were significantly regulated by mating in queens [see Additional file 5]. Thus, there is no apparent bias for the mating process in queens to preferentially regulate nursing-associated or foraging-associated genes.

Previous studies suggest a link between pollen hoarding, reproductive potential, and insulin signaling genes [29]. We did not find any insulin signaling genes in our top 50 most predictive genes in the brains or in the ovaries, nor were insulin signaling genes overrepresented in the top 10 gene ontology lists. However, metabolic genes were significantly overrepresented in the brain gene ontology analysis.

## Discussion

Mating in honey bees causes dramatic changes in behavior (cessation of mating flights, initiation of egg-laying) and physiology (activation of ovary development, increases in vitellogenin synthesis, and changes in pheromone profiles). Here, we demonstrate that these behavioral and physiological transitions are uncoupled in mated honey bee queens, and that they are associated with distinct gene expression patterns in the brains and ovaries. Mated queens *behaved *like virgins (most continued to attempt to take mating flights and none initiated egg-laying), but in terms of physiological parameters, they were in the early stages of the transition to the mated state for pheromone profiles and *vitellogenin *synthesis in their fat bodies, and had partially activated their ovaries. This uncoupling of behavioral and physiological parameters was mirrored in the gene expression patterns of the 50 predictive genes in the brains and ovaries. Hierarchical clustering of the 50 predictive genes in the brains demonstrates that a portion of the variation in brain gene expression is strongly associated with behavioral differences (virgins and mated queens are more similar to each other than to laying queens), while hierarchical clustering of the 50 predictive-genes in the ovaries demonstrated that a portion of the variation in ovary gene expression is associated with physiological differences (mated and laying queens are more similar to each other than to virgin queens). Overall, these results suggest that gene expression in the brains and the ovaries of queens may be uncoupled, such that the physical process of mating may trigger changes in the ovaries, but that the brain may require additional stimuli or a longer amount of time to complete the transition.

### Behavioral Changes

The behavioral observations indicate that there is a cue which triggers queens to switch from taking mating flights to laying eggs. However, while the behavioral switch appears to be unidirectional and permanent, the process that prompts this 'decision' to stop mating remains unclear. Our data suggest that cessation of mating flights is not triggered by the act of mating alone, and is supported by previous studies [30,31]. Moreover, it is unlikely that the cue for queens to cease mating attempts and initiate egg-laying is related to the number of mating flights taken, because half of our observed queens initiated egg-laying after a single mating flight while the other half did not. Instead, our findings suggest that the likely cue inhibiting further mating in a queen is associated with the amount of seminal fluid she receives during her earlier flights [14,32,33].

### Physiological Changes

Interestingly, mating elicits several immediate physiological changes in queens, even if the mating process has not been completed. *Vitellogenin *transcript levels began to increase in the fat bodies of mated queens and are intermediate between virgin and laying queens, demonstrating that levels of this transcript begin to increase even in mated queens. Similarly, in contrast to virgins, mated queens have initiated (though not completed) ovary development, and their pheromone profiles are in the early stages of the transition to the fully mated state. Previous studies suggest that virgin queens treated with carbon dioxide (which stimulates egg-laying) or are instrumentally inseminated eventually initiate egg-laying, though the process is delayed compared to naturally mated queens [34,35]. This suggests that the act of mating will rapidly induce physiological changes, but that these changes are slower in incompletely mated queens.

### Common patterns in the brains and the ovaries

A portion of the brain gene expression pattern correlates with the behaviorally-associated pattern observed. Using a leave-one-out cross-validation, we identified the 50 most-predictive transcripts associated with behavior. When we cluster behavioral groups using these transcripts, this is the pattern we observe. However, if all of the regulated genes were used in a hierarchical clustering analysis, then virgin and laying queens appeared more similar to each other than to mated queens. Based on the principal component analysis, a small proportion of the variation in brain gene expression (3%) matches with the behavioral studies, with virgin and mated queens most similar. 6% of the variation demonstrates that mated queens are the outgroup, showing the same pattern observed with the significant gene-expression patterns. However, the majority of the expression variation (91%) showed no distinct differences among groups. This suggests that the brains of the incompletely mated queens are in a transitional state that is not simply intermediate between the virgin and laying states, and that it is not strongly correlated with either endpoint.

Similarly, when only the 50 most-predictive genes in the ovaries were used for hierarchical clustering, a physiologically-associated pattern emerges that correlates with *vitellogenin *levels and the initiation of ovary development. Again, clustering analysis of the significant gene-expression patterns in the ovaries grouped virgin and laying queens together, with mated queens placed as the outgroup. As in the brains, principal components analysis reveals that only a small part of the observed variation (~3%) shows a pattern associated with behavioral and pheromonal differences, with the virgin and mated queens grouping together. 10% of the variation identifies mated queens as the outgroup, showing the same pattern observed with the significant gene-expression clustering patterns. Again, the majority of the expression variation (83%) showed no distinct differences among groups. This suggests that the ovaries of the mated queens are also in a transitional state (mated queens initiated but did not finish ovary development) that is not simply intermediate between the virgin and laying states, and that it is not strongly correlated with either endpoint.

It is important to note that all mated queens (including those that initiated egg-laying) were allowed only a single mating flight, but that the number of males each queen mated with may have varied during this flight. The laying queens all developed ovaries and initiated egg-laying following this single flight. However, the mated queens did not. The variation observed within mated queens was the greatest for the behavioral observations, pheromone profiles, and ovary development. Perhaps this transition state represents a period of greater flux and more individual variability, and thus may explain why this group appears to be the outgroup in the overall expression profiles of the brains and the ovaries.

### Comparisons to previous studies

Previous studies have examined transcriptional changes in females following mating in *Drosophila *[3,4]. We compared our results from brain transcriptional profiling in honey bee queens to the results published by McGraw et.al. [3], which examined changes in transcript abundance in females shortly after mating. Few genes overlapped between our analysis and this study. However, these results are not surprising for both biological and technical reasons. While there are many similarities in the post-mating behavioral and physiological changes between honey bees and *Drosophila *(such as the initiation of egg-maturation and laying), there are several important differences that would contribute to the lack of overlap in gene expression differences. For example, in *Drosophila*, mating reduces female longevity and also stimulates an immune response [3,4]. Chapman et.al. has proposed that this may be due to sexual conflict and reproductive trade-offs, such that the male seminal proteins stimulate the females to produce as many offspring as possible, to the detriment of the female [1]. In honey bees, sexual conflict between males and females is thought to be reduced, since multiple matings in females is advantageous to colony health, by increasing the genetic diversity of the workers (discussed in [35]). Other differences in the mating systems of the two species may have resulted in the lack of observed similarities in the functional categories of regulated genes. Furthermore, the genes identified by McGraw et.al. resulted from microarray studies using whole bodies as the tissue source. Our analysis focused directly on changes in gene expression in the brains and ovaries. Thus, small changes in the brains or ovaries of the *Drosophila *samples may not have been characterized. Finally, these studies also examined *Drosophila *1–3 hours after mating, while our samples were collected 5 days after mating. Previous studies have demonstrated that post-mating transcriptional changes are highly variable at different time points following mating [36], and may possibly explain the lack of overlap in transcriptional differences between these two studies.

Comparison of our results to previous honey bee microarray studies enables us to examine whether particular aspects of worker physiology or behavior are associated with the mating process in honey bee queens. We compared our gene set to sets of genes associated with worker reproduction [18]. We found little overlap among the genes regulated by mating in queens and these gene sets. There were only two genes that were regulated by reproduction in both workers and queens, suggesting that workers may use a different pathway to initiate egg-laying. Furthermore, in the worker reproduction study, individuals were reared in cages and identified as reproductively active by scoring ovary development. Because they were in cages, these individuals were not able to initiate any form of egg-laying behavior. The queens in this study were reared in colonies and were thus allowed to perform the egg-laying behavior as well as having fully-developed ovaries. Similarly, there was no obvious bias for methoprene (a juvenile hormone analog) regulated [19] genes to be involved in post-mating changes in queens. This is consistent with previous studies assessing the levels of juvenile hormone and effects of methoprene treatment on queens. While juvenile hormone levels are strongly linked to behavioral maturation in workers [19], juvenile hormone does not appear to be strongly associated with mating behavior ([15], reviewed in [21]). We also examined genes known to be regulated by QMP in workers [24]. In workers, QMP elicits a "nurse-like" state, which is associated with high levels of vitellogenin. One might expect genes associated with high vitellogenin levels in workers also to be associated with high vitellogenin levels in queens vs. workers, or in mated queens vs. virgin queens. Indeed, Grozinger et.al. [18] observed that these QMP-regulated genes were similarly regulated in virgin queens relative to workers. However, in our study we did not observe any strong bias to regulate QMP-regulated genes. It is possible that QMP-regulated genes in workers may play another role in the behavior or physiology of queens throughout the mating process.

One emerging theory in the evolution of eusocial behavior is the idea that genes involved in solitary female reproduction may have been co-opted for other processes in worker division of labor [37,38]. If this hypothesis is true, we might expect that genes associated with mating state in queens would also be associated with nursing and foraging behaviors in workers. To test this concept, we compared our list of significantly-regulated genes to genes previously identified by Whitfield et al. (2003) as genes associated with nursing and foraging behavior. Our data indicated that some nursing- and foraging-associated genes are regulated by the mating process (~8% of each), but that there is no particular bias to regulate either nursing-associated or foraging-associated genes throughout the mating process in queens.

## Conclusion

This study has examined whole-genome transcriptional regulation in the brains and the ovaries of virgin, mated, and laying honey bee queens. The post-mating changes in honey bee queens are highly complex. Queens undergo a multitude of behavioral and physiological changes, some of which are immediate and rely either directly on mating as a cue or some other aspect of the mating process to trigger these changes. Other changes are more gradual. We have demonstrated that changes in the behavior and physiology of the groups correlate with the underlying variance observed in transcript abundance in the 50 predictive genes in the brain and the ovaries, and that these changes are associated with either a behaviorally-associated pattern or a physiologically-associated pattern. We have also compared our results to previous studies of mating changes in *Drosophila melanogaster *to identify common biological processes affected by mating, as well as transcripts that may be differentially regulated between these two species due to important differences in natural history. Finally, we used our data to test important theories in the evolution of social behavior, but found no clear relationship between the genes associated with either nursing or foraging behaviors and regulation by mating in queens.

## Methods

### Field Methods

The queens (*Apis mellifera carnica*) were reared at NCSU's Lake Wheeler Honey Bee Research Facility in Raleigh, NC. The source colony used for this experiment was headed by a queen (Glenn Apiaries, Fallbrook, CA) instrumentally inseminated with semen from a single drone [39]. Because male bees (drones) are haploid, the average coefficient of relatedness (*G*) among offspring of such an instrumentally inseminated queen is 0.75. Queens were produced by grafting larvae and reared in queenless colonies for 7 days, according to standard rearing practices [40]. Prior to emergence as adults, queen cells were placed into small mating nucleus colonies with approximately 1,000 workers per colony 2 days before their expected emergence. Two days following emergence, surviving queens were randomly assigned to a group (virgin or mating queens), marked with paint, and returned to their respective colonies. Colony entrances were modified with a Plexiglas-covered runway so that if a queen attempted to fly she could be identified. A queen excluder was placed on the entrance so that the observer could prevent queens in the virgin group from flying and release only those queens in the mating group. For queens > 4 days after emergence, the colony entrances were monitored from 2–6 pm daily and any attempts to fly were recorded. Queens in the mated group were allowed to take multiple orientation flights, but only one mating flight: once a queen returned to the colony with the male's endophallus lodged in her sting chamber, she was no longer allowed to fly, and was confined to the colony as in [33]. Five days after mating, queens were collected on dry ice and stored at -80°C for processing. We separated queens into three groups based on their mating and egg-laying status. Virgin and mated queens were confirmed by examining their spermathecae for stored sperm (see below). In total, there were 6 virgin queens, 5 mated queens, and 4 laying queens used in the behavioral and physiological analyses.

### Spermathecae and ovary dissections and analysis

Dissections were performed on ice in RNAlater solution (Qiagen, Valencia, CA). Pictures were taken of each ovary, and an ovary development score was assigned with the following scale: 1 – no development, 2 – larger ovaries, but ovarioles not clearly visible, 3 – visible ovarioles, no eggs, and 4 – fully developed, large ovarioles, mature eggs [see Additional file 8]. The spermathecae were then removed and stored in 100 μl Kiev buffer for sperm counting (0.3 g D+ glucose, 0.41 g potassium chloride, 0.21 g sodium bicarbonate, 2.43 g sodium citrate per 100 mL deionized, sterile water). Ovaries were then stored in RNAlater at -80°C until further processing. The eviscerated abdomens (cuticle with attached fat bodies) were also stored at -80°C until used for quantitative real time PCR analysis (below). Data analysis was performed in JMP 7.0 (SAS, Cary, NC).

### Sperm Counting

Spermathecae were dissected and stored in 100 μl Kiev buffer at -20°C until sperm counting [33]. The spermathecae were ruptured and contents were diluted in 10 ml of Kiev buffer and immediately counted on a hemocytometer. Counts were performed 5 times each, and an average value was obtained. A nonparametric Kruskall-Wallis rank sums was used in JMP to determine significant differences.

### Quantification of Vitellogenin RNA levels by quantitative real-time-PCR (qRT-PCR)

Fat body samples from individual queens were extracted using an RNeasy RNA extraction kit (Qiagen, Valencia, CA). cDNA was synthesized from 200 ng RNA using the SYBRGreen Master Mix (Applied Biosystems, Foster City, CA). qRT-PCR was performed with an ABI Prism 7900 sequence detector and the SYBR green detection method (Applied Biosystems). The housekeeping gene, *eIFS-8 *was used as a control [24,28]. For each sample, triplicate qRT-PCR reactions were performed and averaged. Quantification was performed using a standard curve generated using genomic DNA. A negative control (cDNA reaction without RT-enzyme) and dissociation curve was used as well. Groups were normalized to one sample for ease of graphical representation, and a nonparametric Kruskall-Wallis rank sums was used in JMP to determine significance. The primer sequences were developed in PrimerExpress (Applied Biosystems) as follows.

#### Vitellogenin

Forward 5'TTGACCAAGACAAGCGGAACT 3'

Reverse – 5' AAGGTTCGAATTAACGATGAAAGC 3'

#### eIFS-8

Forward – 5'TGAGTGTCTGCTATGGATTGCAA3'

Reverse: 5'CGTGGAGTGTTATCGTAAGTAGCAA 3'

### Pheromone Analysis

The queen mandibular glands were dissected on dry ice and immersed in 50μl of diethyl ether containing 0.4 μg/μl of undec-10-enoic acid (as an internal standard) for minimum of 24 hours. A 5 μl of the sample was then removed, dried and resuspended with 10 μl of neat bistrimethylsilyltrifluoroacetamide (BSTFA) [13,14]. The derivatized sample was diluted with hexane (100 μl) and a 2 μl portion was analyzed using gas chromatography on a HP 5890 equipped with capillary column (30 m × 0.25 mm ID. 0.5 μm film thickness) DB-5 (5% diphenyl-95% dimethylsiloxane) column (J&W scientific, Folsom, CA) in splitless mode. Helium was used as the carrier gas at a head pressure of 18 psi (flow rate = 1.3 ml/min). The GC temperature was held at 100°C for 1 min and then increased at 5°C/min to 200°C (5 min), followed by an increase of 10°C/min to 250°C (15 min). Injector and FID temperatures were both set at 250°C.

We extracted the mandibular gland of 6 virgin queens, 4 mated queens and 4 laying queens. To examine differences in chemical profiles related to number of inseminations based on the relative proportion of the chemical compounds, a stepwise discriminant analysis was employed, using all the chemical compounds as per Richard 2007, (Statistica 6.0. StatSoft^® ^Inc, Tulsa, OK).

### Microarray extractions and hybridizations

RNA extraction from brains and ovaries were performed using the PicoPure RNA Isolation kit (Arcturus, CA). 200 ng RNA was amplified using the Amino Allyl MessageAmp II aRNA Amplification Kit (Ambion, Austin, TX). 5 ug of amplified RNA was labeled with Cy3 or Cy5 dye (Amersham Biosciences, Buckinghamshire, UK). Two sets of labeled probe were then hybridized to the whole-genome oligonucleotide arrays supplied from the laboratory of Dr. Gene Robinson (University of Illinois, Urbana-Champaign). Brains and ovaries were hybridized using a loop design with dye-swaps incorporated (n = 4 for each group). Arrays were then scanned using the Axon Genepix 4000B scanner (Molecular Devices, Sunnyvale, CA) using GENEPIX software (Agilent Technologies, Santa Clara, CA).

### Microarray Data Analysis

Spots with intensity less than 300 in two or more arrays were removed from the analysis, and spots with less than 10 observations remaining were also excluded from the data set. One ovary array was excluded from the analysis because the noise to signal ratio was too low.

Expression data was log-transformed and normalized using a mixed-model ANOVA (proc MIXED, SAS, Cary, NC) with the following model:

Y = μ + dye + array + block + dye*array + array*block + ε

where Y is expression, dye is a fixed effect, and array, block, and their interactions are random effects. Detection of significance for differential expression on residuals was performed using a mixed-model ANOVA with the model

Y = μ + behavior + dye + array + dye*array + ε

where y is the residual from the previous model, behavior and dye are fixed effects, and array and dye*array are random effects. P-values were corrected for multiple testing using a false discovery rate < 0.05 (proc MULTTEST, SAS).

"Leave-one-out" cross-validation class prediction was performed in GeneSpring using zero-centered, log_2_-transformed normalized values for individual brains. For the analysis, a single brain was withheld from the data set ("test set") while all 11 remaining brains were used as the "training set" to identify the best predictor genes for mating state. The test brain was classified into the most-likely mating-state based on the expression values of these predictor genes. This test was repeated with each brain being used as the test brain iteratively, until all 12 brains had been tested. 50 predictive genes were calculated from the significantly-regulated gene lists produced by gene-specific ANOVAs.

For graphical purposes, hierarchical clustering was performed in JMP. Trees were drawn and "approximately-unbiased" p-values and bootstrap values were obtained using the pvclust package [41] in R (version 2.5.0). Distance was calculated using the Ward method on a correlation-based dissimilarity matrix.

*Results from the microarrays will be submitted to ArrayExpress as per the standards set forth by the Microarray Gene Expression Data Society*.

### Comparative Analysis

The significant genes from the brain expression dataset were compared to several published datasets [3,18,19,28]. Genes that were present on both our list and the published lists were then used in principal component and clustering analyses in GeneSpring, as well as gene ontology analyses with DAVID [17].

## Authors' contributions

SDA carried out the behavioral, physiological, and molecular genetic studies, participated in the pheromonal analysis, performed the statistical analysis, and drafted the manuscript. FJR participated in the pheromonal study and its statistical analysis. DRT participated in conception and design of the study, participated in behavioral assays, and inseminated the queens. CMG participated in the conception and design of the study, helped to draft the manuscript and coordinated the research. All authors read and approved the final manuscript.

## Supplementary Material

Additional file 1 **Relative quantity of the mandibular gland compounds of virgin (Virgin), mated (M) and laying (L) queens**. The results are presented as relative proportions: tr = traces; + = 0.5–1%; ++ = 1–2%; +++ = 2–5%; ++++ = 6–16%; > = greater than 16%. HOB and dihydroferulic acid differed significantly among groups (Kruskall-Wallis rank sums, p = 0.038 and p = 0.034 respectively). HOB was present in greater concentrations in virgin and mated queens relative to laying (Mann-Whitney U-test, p = 0.01 and p = 0.04 respectively). There was also a higher concentration of dihydroferulic acid in virgin relative to laying queens (Mann-Whitney U-test, p = 0.01). Click here for file

Additional file 2 **50 most-predictive brain genes**. Leave-one-out cross-validation was used to identify the 50 most-predictive transcripts for mating-state in the brains. The 50 genes are listed in this table. Click here for file

Additional file 3 **50 most-predictive ovary genes**. Leave-one out cross-validation was used to identify the 50 most-predictive transcripts for mating-state in the ovaries. The 50 genes are listed in this table. Click here for file

Additional file 4 **Significantly-regulated genes in the brains and the ovaries**. All genes significantly-regulated in the brains and the ovaries with FDR < 0.05 are listed in this table. Click here for file

Additional file 5 **Comparative analyses**. All significant genes identified in the comparative analyses are listed in this table. Click here for file

Additional file 6 **Significant Brain Gene Expression Clustering**. Hierarchical clustering analysis was employed to determine if there was a significant clustering structure among all significantly regulated transcripts in the brains. Virgin and laying queens grouped together with mated queens as the outgroup. This grouping is supported by an "approximately-unbiased" p-value of 100 and a bootstrap support value of 100. Click here for file

Additional file 7 **Significant Ovary Gene Expression Clustering**. Hierarchical clustering analysis was employed to determine if there was a significant clustering structure among all significantly regulated transcripts in the ovaries. Virgin and laying queens grouped together with mated queens as the outgroup. This grouping is supported by an "approximately-unbiased" p-value of 100 and a bootstrap support value of 100. Click here for file

Additional file 8 **Ovary development scale**. Ovaries were assigned a score from 1–4. 1 was completely undeveloped, 4 was completely developed with mature eggs present. Pictures of each of these stages are provided in this figure. Click here for file
