# Supplementary figures and images for: Genomic analysis of post-mating changes in the honey bee queen (Apis mellifera)
Source: BMC Genomics. 2008 May 19;9:232. doi: 10.1186/1471-2164-9-232 (PMC2413142; doi:10.1186/1471-2164-9-232)

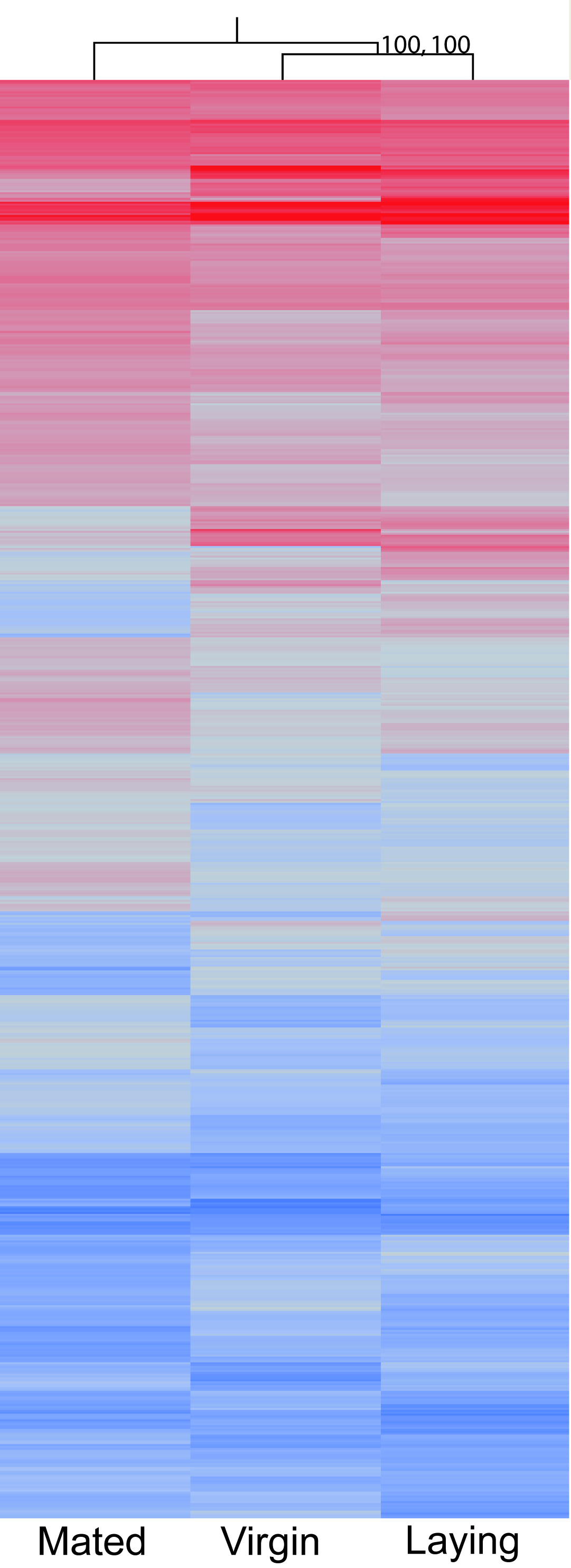

Supplement: Additional file 6 — Significant Brain Gene Expression Clustering. Hierarchical clustering analysis was employed to determine if there was a significant clustering structure among all significantly regulated transcripts in the brains. Virgin and laying queens grouped together with mated queens as the outgroup. This grouping is supported by an "approximately-unbiased" p-value of 100 and a bootstrap support value of 100. [file 1471-2164-9-232-S6.tiff]

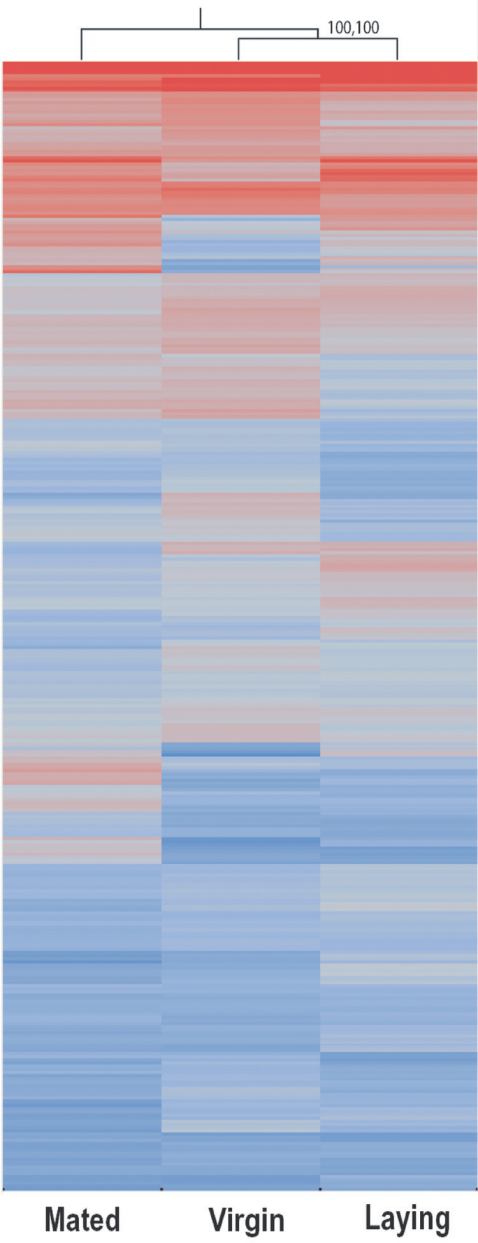

Supplement: Additional file 7 — Significant Ovary Gene Expression Clustering. Hierarchical clustering analysis was employed to determine if there was a significant clustering structure among all significantly regulated transcripts in the ovaries. Virgin and laying queens grouped together with mated queens as the outgroup. This grouping is supported by an "approximately-unbiased" p-value of 100 and a bootstrap support value of 100. [file 1471-2164-9-232-S7.pdf]

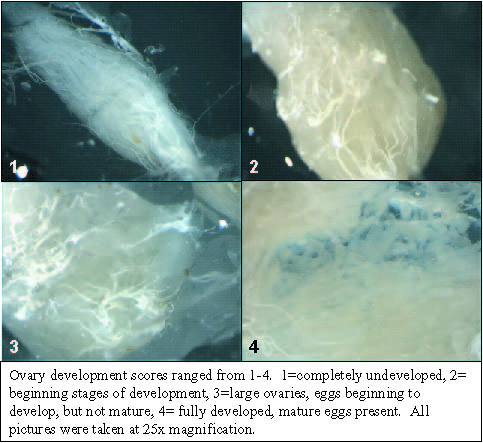

Supplement: Additional file 8 — Ovary development scale. Ovaries were assigned a score from 1–4. 1 was completely undeveloped, 4 was completely developed with mature eggs present. Pictures of each of these stages are provided in this figure. [file 1471-2164-9-232-S8.jpeg]
